# Survival analysis of patients with non-small cell lung cancer who underwent surgical resection following 4 lung cancer resection guidelines

**DOI:** 10.1186/1471-2407-14-422

**Published:** 2014-06-10

**Authors:** Dongsheng Yue, Liqun Gong, Jian You, Yanjun Su, Zhenfa Zhang, Zhen Zhang, Feng Gu, Meng Wang, Changli Wang

**Affiliations:** 1 Department of Lung Cancer, Lung Cancer Center, Tianjin Medical University Cancer Institute and Hospital, Key Laboratory of Cancer Prevention and Therapy, National Clinical Research Center of Cancer, Huan-Hu-Xi Road, Ti-Yuan-Bei, He Xi District, Tianjin 300060, P.R. China

**Keywords:** Non-small cell lung cancer, Resection, Guidelines, Accurate staging, Lymph node dissection

## Abstract

**Background:**

To compare survival of patients with non-small cell lung cancer (NSCLC) who underwent surgical resection and lymph node sampling based on guidelines proposed by the American College of Surgeons Oncology Group (ACOSOG), National Comprehensive Cancer Network (NCCN), the OSI Pharmaceutical RADIANT trial, and the International Association for the Study of Lung Cancer (IASLC).

**Methods:**

Medical records of patients with NSCLC who underwent surgical resection from 2001 to 2008 at our hospital were reviewed. Staging was according to the 7^th^ edition of the AJCC TNM classification of lung cancer. Patients who received surgical resection following the IASLC, ACOSOG, RADIANT or NCCN resection criteria were identified.

**Results:**

A total of 2,711 patients (1803 males, 908 females; mean age, 59.6 ± 9.6 years) were included. Multivariate Cox proportional hazards regression analysis indicated that increasing age, adenosquamous histology, and TNM stage II or III were associated with decreased overall survival (OS). Univariate analysis and log-rank test showed that surgical resection following the guidelines proposed by the IASLC, NCCN, ACOSOG, or RADIANT trial was associated with higher cumulative OS rates (relative to resection not following the guidelines). Multivariate analysis revealed that there was a significant improvement in OS only when IASLC resection guidelines (complete resection) were followed (hazard ratio = 0.84, 95% confidence interval 0.716 to 0.985, *P* = 0.032).

**Conclusions:**

Surgical resection following the criteria proposed by IASLC, NCCN, ACOSOG, or the RADIANT trial was associated with a higher cumulative OS rate. However, significant improvement in OS only occurred when IASLC resection guidelines were followed.

## Background

Lung cancer is the leading cause of cancer-related mortality in men and women in the United States and throughout the world [1]. In the United States, an estimated 228,190 new cases of lung cancer are expected in 2013, accounting for about 14% of cancer diagnoses [2]. Further, an estimated 159,480 deaths due to lung cancer are expected to occur, accounting for approximately 27% of all cancer deaths [2]. Non-small cell lung carcinoma (NSCLC) accounts for approximately 85% of all lung cancers in the United States [2]. In China, lung cancer has the highest incidence (53.57/100,000 in 2009) and mortality (45.57/100,000 in 2009) among all cancers [3]. Most lung carcinomas are diagnosed at an advanced stage and, as such, have a poor prognosis. The 5-year relative survival rates vary with the stage of disease at diagnosis, with reported rates being 49% for local disease, 16% for regional disease, and 2% for distant disease [4].

Accurate staging is a critical aspect of the diagnostic work-up of patients with NSCLC, with disease stage influencing decisions regarding the type and timing of treatments [5]. Surgical resection remains the primary treatment for patients with stage I and II NSCLC [6,7]. The role of surgery for stage III disease is controversial, while patients with stage IIIB or IV tumors are rarely surgical candidates [7]. Cerfolio et al [8] reported that about 14% NSCLC patients were clinically over-staged (benign nodules) and 32% were clinically under-staged (most from nonimaged N2 disease). Based on their report, only 56% of patients clinically staged as having stage I NSCLC had pathologic stage I disease while 6.8% of patients clinically staged as having greater than stage I had pathologic stage I disease, suggesting that complete thoracic lymphadenectomy improve the staging of patients with NSCLC.

Curative treatment of early stage NSCLC requires good quality surgical resection (GQR); however, some issues of contention exist. On the one hand, the findings from a previous study suggest that the degree of compliance with national recommendations for GQR is poor, and that the majority of curative-intent resections of early stage NSCLC did not achieve GQR standards [9]. On the other hand, there is no consensus as to what constitutes a minimally acceptable degree of surgical resection [9]. GQR guidelines have been proposed by a number of groups, including the International Association for the Study of Lung Cancer (IASLC) [10], American College of Surgeons Oncology Group (ACOSOG) [11], National Comprehensive Cancer Network (NCCN) [12], and OSI Pharmaceutical RADIANT trial [9,12] (note: the RADIANT trial is an ongoing trial of erlotinib after surgery with or without adjuvant chemotherapy in patients with NSCLC epidermal growth factor receptor positive tumors [12]). The IASLC, NCCN, ACOSOG, and RADIANT resection guidelines all require negative surgical margins as determined microscopically, but differ in the degree of lung resection and lymph node sampling and resection [7,9-12]. Lymph node status is closely associated with prognosis in patients with NSCLC and is an important component of the lung cancer staging classification system [13-15]. However, the extent of lymph node removal required and the impact of mediastinal node removal on survival are controversial [16]. The NCCN, ACOSOG, and RADIANT resection guidelines require certain degrees of lymph node sampling [7,11,12]. However, the IASLC guidelines indicate that complete resection of lymph nodes should be performed instead of lymph node sampling [15].

The major differences between the IASLC, NCCN, ACOSOG, and RADIANT resection guidelines are the degree of lung resection and lymph node sampling versus resection [7,10-12]. The ACOSOG requires examination of station 10 (hilar lymph nodes) in addition to other N1 nodes, and at least 1 lymph node from stations 2, 4, and 7 for right-sided tumors or stations 5, 6, and 7 for left-sided tumors [11]. The NCCN criteria require N1 and N2 lymph node resection and mapping with sampling from a minimum of 3 N2 stations [7]. The RADIANT study criteria requires at least 2 mediastinal stations be sampled, though the exact stations are not specified and N1 sampling is not required [12]. Instead of sampling, the IASLC requires systematic nodal dissection or lobe-specific systematic nodal dissection, no extracapsular involvement of the tumor, and the highest mediastinal node removed must be negative [10]. Thus, surgical resection that follows the IASLC GQR guidelines would also meet the criteria proposed by other GQR guidelines.

The purpose of this study was to compare survival in patients with NSCLC by the resection guideline followed (all four aforementioned guidelines were included to assess whether differences in criteria affect outcomes). The factors associated with survival and the cumulative survival rate were compared. Also, since more surgeons performed complete lymphadenectomy without lymph node sampling (IASLC guidelines) after 2005, we aimed to determine whether this change in surgical procedure would affect the survival of the NSCLC patients.

## Methods

### Participants

In this study, the medical records of patients with NSCLC who underwent surgical resection from 2001 to 2008 at Tianjin Medical University Cancer Institute and Hospital, were reviewed. This study was approved by the Institutional Review Board of Tianjin Medical University Cancer Institute and Hospital. Due to the retrospective nature of the study, the requirement for informed patient consent was waived. Patients were included in the study if they had a diagnosis of NSCLC confirmed by pathological tissue examination and underwent surgical resection. Exclusion criteria were: 1) Staging data was not available; 2) Data to determine which surgical resection guideline followed was not available; (3) Received radiotherapy before resection; (4) Secondary malignancy identified within 5 years after resection; (5) Incomplete follow-up data.

### Data collection

Data extracted from the medical records included age, gender, smoking history, surgical procedure, surgical margin status, histological diagnosis, T stage, N stage, TNM stage, number of lymph node stations examined, number of lymph nodes removed, and treatments (i.e., surgical resection, adjuvant chemotherapy, radiotherapy or immunotherapy). Patients were restaged according to the 7^th^ edition of the American Joint Committee on Cancer (AJCC) TNM classification of lung cancer [10]. Patients who received surgical resection following the IASLC, ACOSOG, RADIANT, or NCCN resection criteria were identified. In addition, patients were divided into 2 time periods: Period I: those who received surgery during the period from 2001 to 2004, when lymph node sampling was performed by most surgeons at our hospital; Period II: those who received surgery during the period from 2005 to 2008, when more surgeons performed complete resection following the definition proposed by the IASLC [10]. The IASLC requires: that the free resection margins be proven microscopically; systematic nodal dissection or lobe-specific systematic nodal dissection; no extracapsular nodal extension of the tumor; and that the most distant nodal stations (the highest in the superior paratracheal and the lowest in the pulmonary ligament) are negative [10].

### Statistical analysis

Demographics and clinical characteristic of the subjects are summarized as mean ± standard deviations (SD) for continuous variables and number (%) for categorical variables. Univariate and multivariate Cox proportional hazards regression analyses were carried out to identify factors having a significant impact on overall survival (OS). Significant variables (*P* < 0.05) in univariate Cox proportional hazards regression analysis were entered into multivariate analysis. The associated results are reported as hazard ratios (HRs) with 95% confidence intervals (CIs). Kaplan-Meier curves of OS are presented for the cumulative survival rates versus follow-up time for patients in whom the 4 lung cancer resection guidelines were or were not followed. The log-rank test was performed to identify differences in OS between patients in which the 4 resection guidelines were or were not followed. The cumulative 6-month and 1-, 3-, and 5-year survival rates with corresponding 95% CIs were also determined for patients in which the 4 resection criteria were or were not followed. All statistical assessments were 2-tailed. A value of *P* < 0.05 was considered to indicate statistical significance. Statistical analyses were performed using SPSS 18.0 statistics software (SPSS Inc, Chicago, IL, USA).

## Results

### Demographic and clinical characteristics

The demographic and clinical characteristics of the subjects are shown in Table 1. The records of 3,346 patients were reviewed, and a total of 2,711 NSCLC patients (1803 males, 908 females) with a mean age of 59.6 ± 9.6 years were included in the analysis after applying the inclusion and exclusion criteria. Of the included patients, 83.1% received lobectomy. The majority of patients (50.4%) had a histological diagnosis of squamous cell carcinoma and adenocarcinoma was second most common (34.1%). The majority of patients (42.5%) were at stage I and those at stage III followed (32.9%). Fewer than 6 lymph node stations were examined in almost 40% of patients. Twenty or more lymph nodes were removed in about 40% of patients and fewer lymph nodes removed in the remaining patients. The majority of patients received adjuvant chemotherapy after the surgical treatment and fewer patient received radiotherapy and immunotherapy.

**Table 1:** Demographic and clinical characteristics of subjects (N = 2711)

**Age (y)**	**59.6 ± 9.6**
Gender	
Male	1803 (66.5)
Female	908 (33.5)
Smoking history	
Smoker	1722 (63.5)
Never	989 (36.5)
Surgical procedure	
Segmentectomy	65 (2.4)
Lobectomy	2254 (83.1)
Pneumonectomy	373 (13.8)
Extended	19 (0.7)
Surgical margin status	
Positive	130 (4.8)
Negative	2581 (95.2)
Histology	
Squamous	1365 (50.4)
Adenocarcinoma	924 (34.1)
Large cell	33 (1.2)
Adenosquamous	159 (5.9)
Other	230 (8.5)
T stage	
T1/T2	2354 (86.8)
T3/T4	357 (13.2)
N stage	
N0	1544 (57.0)
N1/N2	1167 (43.0)
AJCC TNM Stage	
IA	558 (20.6)
IB	589 (21.7)
II A	444 (16.4)
IIB	229 (8.4)
III	891 (32.9)
Lymph node stations examined	
< 6 group	1040 (38.4)
≥ 6 group	1671 (61.6)
Number of lymph nodes removed	
< 10	689 (25.4)
10-20	943 (34.8)
≥ 20	1079 (39.8)
Adjuvant chemotherapy	
Yes	1333 (49.2)
No	1378 (50.8)
Radiotherapy	
Yes	427 (15.8)
No	2284 (84.2)
Immunotherapy	
Yes	224 (8.3)
No	2487 (91.7)

Data are presented as mean ± standard deviation (SD) or number (percentage).

^*^1151 patients were classified into stage I, 669 in stage II, and 891 in stage III.

### Cox proportional hazards regression analysis

The results of the Cox proportional hazards regression analysis of potential factors affecting OS are shown in Table 2. Univariate analysis revealed that age, surgical procedure (pneumonectomy vs. segmentectomy), positive surgical margins, histological diagnosis (adenocarcinoma vs. squamous and squamous vs. adenosquamous), TNM stage II/III, < 6 lymph node stations examined, number of lymph nodes removed (<10 vs. ≥ 20), and a lack of radiotherapy were associated with decreased OS (all *P* < 0.05). In contrast, resection in accordance with IASLC, ACOSOG, RADIANT, and NCCN guidelines (vs. resection not in accordance with these guidelines) was associated with improved OS (*P* < 0.001).

**Table 2:** Univariate and multivariate Cox proportional hazards regression analysis of factors affecting overall survival (N = 2711)

	**Univariate**		**Multivariate**	
**Variables**	**HR (95% CI)**	***P* value**	**HR (95% CI)**	***P *** **value**
Age (y)	1.008 (1.002, 1.013)	0.006*	1.014 (1.008, 1.020)	<0.001*
Gender				
Female	reference			
Male	1.017 (0.909, 1.138)	0.774		
Smoking history				
Never	reference			
Smoker	1.061 (0.950, 1.185)	0.293		
Surgical procedure		<0.001*		<0.001*
Segmentectomy	reference		reference	
Lobectomy	1.002 (0.709, 1.418)	0.989	0.975 (0.672, 1.413)	0.892
Pneumonectomy	1.451 (1.006, 2.092)	0.047*	1.338 (0.901, 1.986)	0.149
Extended	1.668 (0.892, 3.119)	0.109	1.533 (0.804, 2.921)	0.194
Surgical margin status				
Negative	reference		reference	
Positive	1.612 (1.298, 2.004)	<.001*	1.200 (0.883, 1.632)	0.243
Histology		0.006*		0.034*
Squamous	reference		reference	
Adenocarcinoma	0.884 (0.785, 0.997)	0.044*	1.000 (0.883, 1.132)	0.999
Large cell	0.912 (0.547, 1.522)	0.726	1.002 (0.600, 1.674)	0.993
Adenosquamous	1.327 (1.067, 1.651)	0.011*	1.347 (1.080, 1.680)	0.008*
Other	1.084 (0.896, 1.312)	0.409	1.208 (0.994, 1.467)	0.057
T stage				
T1/T2	reference			
T3/T4	1.646 (1.430, 1.895)	<0.001*		
N stage				
N0	reference			
N1/N2	1.697 (1.526, 1.887)	<0.001*		
AJCC TNM Stage		<0.001*		<0.001*
I	reference		reference	
II	1.612 (1.405, 1.850)	<0.001*	1.571 (1.366, 1.808)	<0.001*
III	2.172 (1.917, 2.461)	<0.001*	2.101 (1.835, 2.405)	<0.001*
Lymph node stations examined				
≥ 6 group	reference		reference	
< 6 group	1.197 (1.075, 1.333)	0.001*	1.199 (0.771, 1.863)	0.421
Number of lymph nodes removed		<0.001*		0.075
≥ 20	reference		reference	
10-20	0.954 (0.842, 1.082)	0.463	0.935 (0.819, 1.067)	0.318
< 10	1.264 (1.109, 1.441)	<0.001*	1.125 (0.943, 1.343)	0.190
Followed IASLC guidelines				
Yes	0.715 (0.641, 0.797)	<0.001*	0.840 (0.716, 0.985)	0.032*
No	reference		reference	
Followed ACOSOG guidelines				
Yes	0.816 (0.733, 0.908)	<0.001*	1.088 (0.389, 3.045)	0.873
No	reference		reference	
Followed RADIANT guidelines				
Yes	0.785 (0.696, 0.885)	<0.001*	0.887 (0.735, 1.071)	0.213
No	reference		reference	
Followed NCCN guidelines				
Yes	0.814 (0.732, 0.906)	<0.001*	1.089 (0.422, 2.811)	0.860
No	reference		reference	
Adjuvant chemotherapy				
Yes	0.989 (0.890, 1.099)	0.835		
No	reference			
Radiotherapy				
Yes	0.768 (0.671, 0.877)	<0.001*	0.915 (0.797, 1.049)	0.203
No	reference		reference	
Immunotherapy				
Yes	1.063 (0.83, 1.281)	0.517		
No	reference			

ACOSOG, American College of Surgeons Oncology Group; IASLC, International Association for the Study of Lung Cancer (IASLC); NCCN, National Comprehensive Cancer Network; RADIANT, OSI Pharmaceutical’s RADIANT study; HR, hazard ratio; CI, confidence interval.

Variables with a significant association with overall survival in univariate analysis (*P* < 0.05) were selected and put into the multivariate analysis.

Note: Because T stage, N stage, and TNM stage are collinear, only TNM stage was included in the multivariate Cox proportional hazards regression analysis.

*Significant risk factor, *P* < 0.05.

Variables with a significant association (*P* < 0.05) in univariate Cox proportional hazards regression analysis were included in the multivariate analysis, which revealed that advanced age, adenosquamous histology and TNM stages II or III were associated with decreased OS. Use of IASLC resection guidelines (complete resection) was associated with increased OS in comparison to not using IASLC guidelines.

### Kaplan-Meier curves of OS

**Figure 1:**
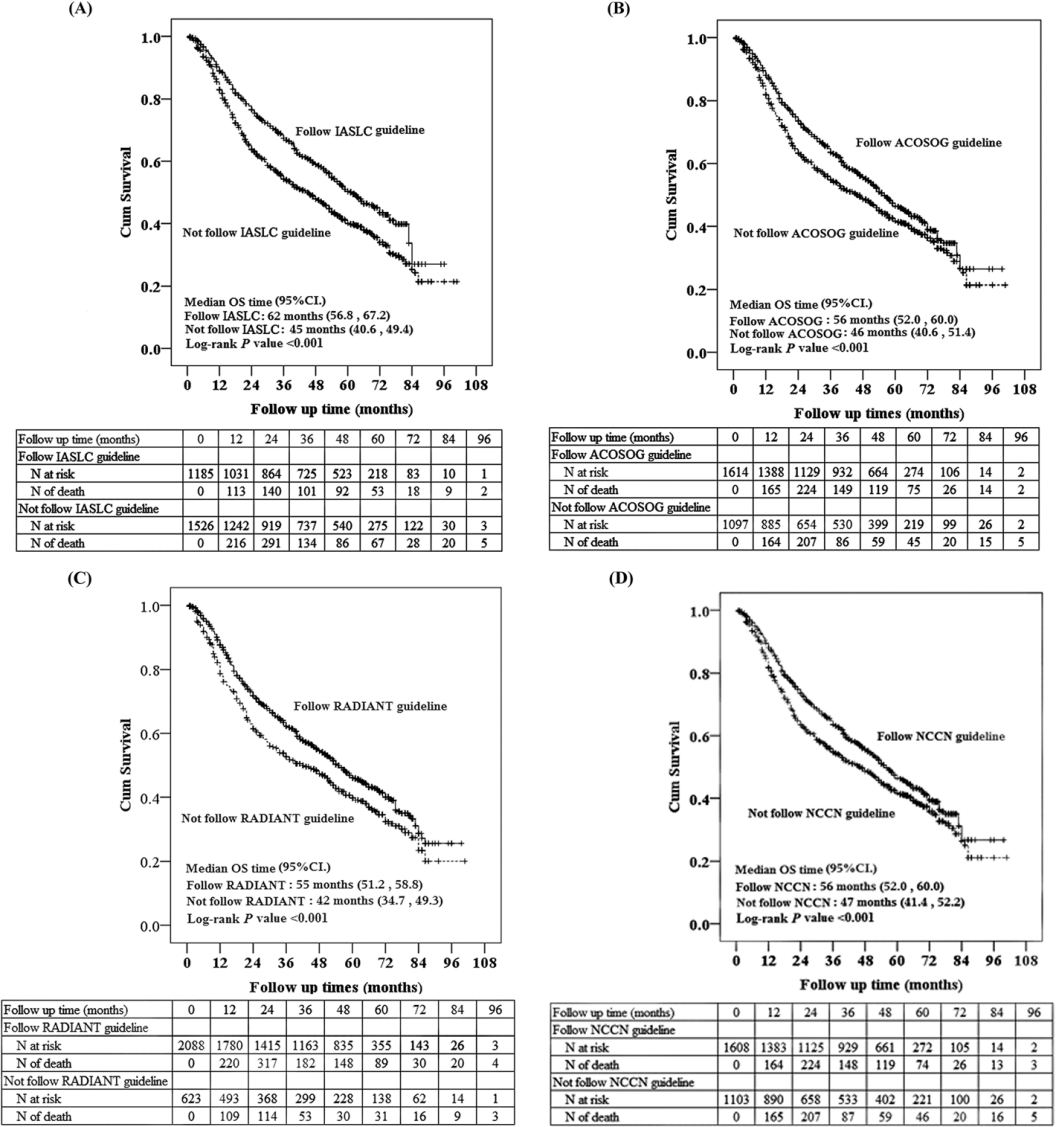
**Kaplan-Meier curves of overall survival (OS) for patients in which the (A) IASLC, (B) ACOSOG, (C) RADIANT, and (D) NCCN GQR guidelines were or were not followed (+indicates censored cases).** The log-rank test showed that OS was significantly different between patients in which the resection guidelines were followed compared with those in which the guidelines were not followed (all *P* < 0.001).

Patients’ estimated median survival time was 50 months and the cumulative 6 month and 1-, 3-, and 5-year survival rates were 95%, 86%, 60% and 45%, respectively (Table 3). The cumulative survival rates with corresponding 95% CIs for patients in which the 4 surgical resection guidelines were or were not followed are also summarized in Table 3. Figure 1 shows the Kaplan-Meier curves for the cumulative survival rates versus follow-up time for the entire cohort of patients in which the 4 resection guidelines were or were not followed. OS was significantly longer for patients for whom the guidelines were followed compared to patients in whom the guidelines were not followed (all *P* < 0.05) (Figure 1).

**Table 3:** Cumulative survival rates of patients in which the 4 lung cancer resection guidelines were or were not followed

	**1-year**	**3-year**	**5-year**	***P***
Overall	0.86 (0.84, 0.87)	0.60 (0.58, 0.62)	0.45 (0.42, 0.47)	
IASLC guidelines				<0.001*
Yes	0.90 (0.88, 0.92)	0.69 (0.66, 0.71)	0.51 (0.48, 0.54)	
No	0.85 (0.84, 0.87)	0.53 (0.58, 0.58)	0.41 (0.38, 0.44)	
ACOSOG guidelines				<0.001*
Yes	0.88 (0.86, 0.9)	0.64 (0.61, 0.66)	0.47 (0.44, 0.49)	
No	0.82 (0.79, 0.84)	0.55 (0.52, 0.58)	0.42 (0.38, 0.45)	
RADIANT guidelines				<0.001*
Yes	0.88 (0.86, 0.89)	0.62 (0.6, 0.64)	0.46 (0.43, 0.49)	
No	0.79 (0.75, 0.82)	0.53 (0.49, 0.57)	0.40 (0.35, 0.44)	
NCCN guidelines				<0.001*
Yes	0.88 (0.86, 0.90)	0.64 (0.61, 0.66)	0.47 (0.44, 0.49)	
No	0.82 (0.79, 0.84)	0.55 (0.52, 0.58)	0.42 (0.38, 0.45)	

ACOSOG, American College of Surgeons Oncology Group; IASLC, International Association for the Study of Lung Cancer (IASLC); NCCN, National Comprehensive Cancer Network; RADIANT, OSI Pharmaceutical’s RADIANT study; CI, confidence interval.

Results are presented as cumulative survival rate (0.0-1.0) with corresponding 95% CI.

*Significant difference between Yes/No follow guidelines, *P* < 0.05.

**Figure 2:**
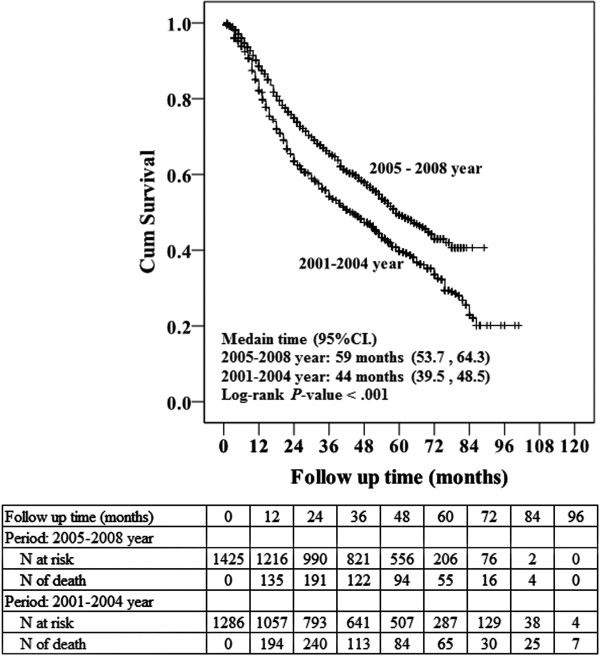
**Kaplan-Meier curves of overall survival (OS) for time period 2001 to 2004 (more surgeons performed lymph node sampling) (blue line) and 2005 to 2008 (more surgeons performed complete resection) (green line) (+indicates censored cases). ***P* < 0.001 indicates OS was significantly different between the 2 periods.

Table 4 presents the number of patients for whom the 4 resection guidelines were followed during the periods from 2001 to 2004 (29.6%) and from 2005 to 2008 (56.4%), representing a significant increase between the two periods. Note: a greater proportion of curative-intent surgical resections complied with GQR guidelines (IASLC, NCCN, ACOSOG, or RADIANT) between 2005 to 2008. The Kaplan-Meier curves of the cumulative survival for the periods from 2001 to 2004 and 2005 to 2008 are shown in Figure 2. The cumulative survival rate was greater in the period from 2005 to 2008 than in the period from 2001 to 2004 (log-rank test, *P* < 0.05). The total number of patients in whom the 4 resection guidelines were followed includes 43.7% following IASLC resection guidelines, 59.5% following ACOSOG guidelines, 77% following RADIANT guidelines and 59.3% following NCCN guidelines (Figure 2).

**Table 4:** Patients in which the 4 lung cancer resection guidelines were followed in the 2 periods

**Guideline followed**	**Total (N = 2711)**	**2001-2004 (n = 1286)**	**2005-2008 (n = 1425)**	***P***
IASLC	1185 (43.7)	381 (29.6)	804 (56.4)	<0.001*
ACOSOG	1614 (59.5)	560 (43.5)	1054 (74.0)	<0.001*
RADIANT	2088 (77.0)	811 (63.1)	1277 (89.6)	<0.001*
NCCN	1608 (59.3)	555 (43.2)	1053 (73.9)	<0.001*

Data are presented as number (percentage) of patients following the guidelines. Differences between the 2 periods were compared using the Pearson Chi-square test.

*Significant difference between the 2 time periods, *P* < 0.05.

## Discussion and conclusions

This study showed that surgical resection of NSCLC without following the GQR guidelines proposed by the IASLC, NCCN, ACOSOG, or RADIANT trial did affect the OS among our NSCLC patients between 2001 and 2008. However, multivariate Cox analysis indicated that only following IASLC resection guidelines (complete resection) was a favorable factor for OS (HR = 0.840, 95% CI 0.716-0.985, *P* = 0.032). Our observation is not consistent with that reported by Allen et al. [9], who found that 3-year survival was not significantly higher among patients who received resection in accordance with the GQR guidelines compared with those who did not receive resection in accordance with the guidelines. The difference between our finding and that reported by Allen et al. [9] may be related study population differences, in particular, the difference in the percentage of patients with stage I disease. In the study reported by Allen et al. [9], 69% of patients had stage I disease, whereas in our study, only 42.5% of patients had AJCC stage I disease. Hence, patients with > stage I disease may derive greater benefit from surgical resection that complies with the GQR guidelines.

The impact of mediastinal node removal and the extent of lymph node removal on survival are still being debated [9]. Some long-term follow-up data suggest that the 5-year survival rate following complete resections is unaffected by the nodal strategy, with reported rates of 45% with complete mediastinal lymphadenectomy and 43% when sampling was performed (*P* = 0.18) [10]. Izbicki et al. [17] conducted a randomized controlled trial to compare the survival between mediastinal lymph node sampling and systematic mediastinal lymphadenectomy. After a median follow-up of 47 months, it was found that systematic mediastinal lymphadenectomy did not statistically improve survival in the overall group of patients (34.2% vs. 45.2%), although the rate of recurrence tended to be reduced among patients who underwent complete lymphadenectomy. Allen et al. [18] also conducted a randomized trial comparing lymph node sampling versus mediastinal lymph node dissection for early stage lung cancer and found that there was no association between the type of resection and mortality. However, Jonnalgadda et al. [19] performed an analysis of the SEER database and found that the number of positive lymph nodes was an independent prognostic factor of survival in patients with N1 NSCLC, suggesting that sampling and removing the positive lymph nodes might improve survival. Verhagen et al. [20] reported that a complete lymph node dissection according to the European Society of Thoracic Surgery guidelines for intraoperative lymph node staging in patients with NSCLC was only performed in 4% of patients. A Cochrane review by Manser et al. [21] concluded that lesion resection combined with complete mediastinal lymph node dissection was associated with a small to modest improvement in survival compared with systematic sampling of mediastinal nodes in patients with stage I to IIIA NSCLC. Results of the multivariate regression analysis of our data showed that following the IASLC resection guidelines (complete resection) was associated with improved OS. This finding is consistent with that reported by Manser [21]. Additionally, in our study, OS was significantly better in the period from 2005 to 2008 than in the period from 2001 to 2004. Furthermore, the number of resections that met the IASLC, NCCN, ACOSOG, and RADIANT resection criteria was significantly greater in the period from 2005 to 2008, highlighting the importance of the quality of surgical resection.

Our study has some limitations that should be mentioned. The first limitation is the retrospective nature of the analysis, which means we cannot make an evidence-based conclusion that complete resection provides a survival advantage. The second limitation is the arbitrariness of using the year 2005 as a cut-off. However, in fact, the definition of complete resection was published by the Complete Resection Subcommittee of IASLC in 2005 [10], which encouraged surgeons to change the surgical procedure for NSCLC.

In summary, the OS rate of patients with early stage NSCLC can be increased following GQR as defined by the IASLC, NCCN, ACOSOG, and RADIANT trial. More surgeons routinely performed complete lymphadenectomy and there was increased compliance with GQR guidelines after 2005 at our institution. This may be one of the reasons for the improvement in OS of patients with early stage NSCLC in recent years.

## Abbreviations

ACOSOG: American College of Surgeons Oncology Group; cis: Confidence intervals; GQR: Good quality surgical resection; HR: Hazard ratios; IASLC: International Association for the Study of Lung Cancer; NCCN: National Comprehensive Cancer Network; NSCLC: Non-small cell lung cancer; OS: Overall survival.

## Competing interests

The authors declare that they have no competing interests.

## Authors’ contributions

DSY and CLW put forward the study concepts and guaranteed the integrity of the entire study. DSY performed the literature research. LQG,JY,YJS, ZFF, ZZ,FG, and MW acquired the data. ZZ and MW analyzed the data and statistics. DSY and CLW prepared and edited the manuscript. LQG, JY, YJS, ZZ,FG,CLW and YHW revised the manuscript. All authors read and approved the final manuscript.

## Pre-publication history

The pre-publication history for this paper can be accessed here:

http://www.biomedcentral.com/1471-2407/14/422/prepub
